# Exosome-Mediated Insulin Delivery for the Potential Treatment of Diabetes Mellitus

**DOI:** 10.3390/pharmaceutics13111870

**Published:** 2021-11-05

**Authors:** Belén Rodríguez-Morales, Marilena Antunes-Ricardo, José González-Valdez

**Affiliations:** School of Engineering and Science, Tecnologico de Monterrey, Av. Eugenio Garza Sada 2501 Sur, Monterrey 64849, NL, Mexico; rdguezmorales.belen@gmail.com

**Keywords:** exosomes, insulin delivery, HepG2, HDFa, RIN-m

## Abstract

Exosomes are extracellular nanovesicles between 30 and 150 nm that serve as essential messengers for different biological signaling and pathological processes. After their discovery, a wide range of applications have been developed, especially in therapeutic drug delivery. In this context, the aim of this work was to test the efficiency of exosome-mediated human insulin delivery using exosomes extracted from three different cell lines: hepatocellular carcinoma (HepG2); primary dermal fibroblasts (HDFa) and pancreatic β cells (RIN-m); all are related to the production and/or the ability to sense insulin and to consequently regulate glucose levels in the extracellular medium. The obtained results revealed that the optimal insulin loading efficiency was achieved by a 200 V electroporation, in comparison with incubation at room temperature. Moreover, the maximum in vitro exosome uptake was reached after incubation for 6 h, which slightly decreased 24 h after adding the exosomes. Glucose quantification assays revealed that exosome-mediated incorporation of insulin presented significant differences in HDFa and HepG2 cells, enhancing the transport in HDFa, in comparison with free human insulin effects in the regulation of extracellular glucose levels. No significant differences were found between the treatments in RIN-m cells. Hence, the results suggest that exosomes could potentially become a valuable tool for stable and biocompatible insulin delivery in diabetes mellitus treatment alternatives.

## 1. Introduction

Intercellular communication is essential to maintain cellular and tissue functions and homeostasis in multicellular organisms. This coordinated cellular activity is mediated by direct cell–cell contact or through the transfer of secreted molecules. Even in the most essential pathways, a wide range of cells release different types of vesicles, such as microvesicles (MVs) and a smaller nanosized population called exosomes. The release of exosomes to the extracellular medium occurs by inward budding and fusion of multivesicular bodies (MVBs) with the plasma membrane [1,2]. These extracellular vesicles (EVs) play an important role as carriers for the transfer of proteins, lipids, DNA and RNA between cells [1].

Exosomes have been successfully isolated and purified from cell cultures and body fluids [3]. Specifically, exosomes are membrane vesicles with an average size from 30 to 150 nm and, structurally, their membrane is composed of proteins and lipids [4]. Moreover, exosomes normally contain specific proteins in their membrane, such as CD9, CD63, CD81 and CD82 tetraspanins, which are often used as markers for exosomal detection [5]. Exosomal tetraspanin complexes facilitate vesicular fusion and fission [6], probably modulated by cellular integrins [7]. The biological functions of EVs directly depend on their ability to interact and deliver their content inside the target cell. Exosomes are involved in diverse biological functions, such as tumor progression by promoting angiogenesis and cancerous cell migration [8], delivery of antigenic molecules during the immune response [9], membrane exchange between cells and miRNA transfer in regulation pathways [10].

Consequently, the discovery of different types of exosomes, exosomal cargo, ways of transportation and biological functions for these EVs determined that exosomes act as essential messengers for numerous biological signaling processes, as well as in a large group of pathological processes [11], some of them as complex as metabolic pathways. For example, Tan et al. collected mesenchymal stem cell-derived (MSC) exosomes and introduced them to a mouse model treated with different xenobiotic compounds, concluding that MSC-derived exosomes can produce hepatoprotective effects in drug-induced damaged liver cells, through the activation of regeneration and proliferation signals [12]. Moreover, it is known that in Type 2 diabetes patients, miRNAs in pancreas-derived exosomes are implicated in the survival and apoptosis of pancreatic β-cells [13].

Diabetes mellitus (DM), commonly known as diabetes, is a group of metabolic disorders that affect millions of people worldwide. So far, the only effective treatment consists of daily subcutaneous injections of insulin [14]. Nevertheless, insulin is a therapeutic peptide hormone characterized by rapid degradation by the enzymes in the gastrointestinal tract and although oral formulations are available, insulin bioavailability falls to less than 2% after administration [15]. In this sense, even when drug delivery opportunities are available in the oral administration pathways, subcutaneous delivery strategies are also available for optimization. Therefore, the search for novel subcutaneous strategies to deliver it focus on protecting insulin against proteolytic degradation and, consequently, conserving its native structure and biological function.

In previous studies, liposomes have been investigated as potential drug delivery systems for peptide and protein drugs, such as bovine serum albumin (BSA), which has been successfully loaded in cationic liposomes [16]. Furthermore, it is now known that human insulin conserves its biological activity after encapsulation in poly(isobutylcyanoacrylate) nanocapsules obtained by interfacial polymerization [17]. Therefore, the use of nanocarriers such as polymeric liposomes seem to be a stable and effective delivery alternative for a variety of therapeutic applications, such as gene therapy, immunotherapy, and diagnosis. Despite this, several synthetic non-natural nanocarriers do not reach their goal, and they often produce some toxicity in the recipient cell population or are eliminated by the host’s immune system [18]. Consequently, exosomes, which present similar properties to liposomes in terms of their enclosing lipidic bilayer plus their embedded signal proteins and molecules in their surface, might represent an attractive and efficient insulin delivery system.

In this context, this work was conceived to test and corroborate the potential use of exosomes for encapsulating and delivering insulin in vitro. The cellular uptake of insulin-loaded exosomes was determined, quantified, and visualized by labeling human insulin with fluorescein isothiocyanate (FITC). Therefore, the final aim was to determine the loading efficiency of FITC-labeled human insulin inside hepatocellular carcinoma, primary dermal fibroblasts, and pancreatic islet cell tumor-derived exosomes, and, subsequently, to evaluate exosome-mediated incorporation of insulin and its effect on the regulation of glucose levels compared with free human insulin in the same cell lines exposed to a high-glucose medium. It should be noted that even when insulin exerts its glucose-regulating function extracellularly, a delivery system such as exosomes might provide a suitable option for its release near the target tissue, considering that part of the loaded molecule will be internalized, as will be shown in this work.

## 2. Materials and Methods

### 2.1. Cell Lines and Reagents

Hepatocellular carcinoma (HepG2) (ATCC HB-8065™), primary dermal fibroblasts (HDFa) (ATCC PCS-201-012™) and pancreatic islet cell tumor (RIN-m) (ATCC CRL-2058™) cell lines were obtained from the American Type Cell Collection (ATCC, Manassas, VA, USA). Dulbecco’s modified Eagle medium: Nutrient Mixture F-12 (DMEM-F12) at pH 7.4, fetal bovine serum (FBS), phosphate-buffered saline (1×) solution at pH 7.4 (PBS) and trypsin 0.25% EDTA (1×) were purchased from Gibco (Grand Island, NY). FITC-labeled human insulin, human recombinant insulin, fluorescein-5-isothiocyanate (FITC), dimethyl sulfoxide (DMSO), D-(+)-glucose (≥95.5%), 3,5-dinitrosalicylic acid (DNS), potassium sodium tartrate tetrahydrate, potassium disulfide and phenol were purchased from Sigma-Aldrich (Saint Louis, MO, USA).

### 2.2. Cell Culture

All cell lines were maintained independently with DMEM-F12 (DMEM) medium at pH 7.4, supplemented with 10% exosome-free FBS (Gibco, Grand Island, NY, USA) at 37 °C and a 5% CO_2_ humidified atmosphere. For subculturing all cell lines, approximately $1\times 10^{6}$ cells were placed in 100 mm × 20 mm tissue-culture treated culture dishes (Corning, NY, USA) for between 24 and 48 h until they reached an approximate confluence of 80%. During the subculturing procedures, the maintenance medium which contained the released exosomes was collected and replaced with 5–6 mL of fresh medium as part of the subculture process. The maintenance medium was stored at −20 °C until use.

### 2.3. Exosome Isolation

The recollected exosome-enriched maintenance medium was first clarified to remove cells and debris suspended in the medium. The clarification process consisted of centrifugation at 2100× *g* for 30 min in an Allegra 64R centrifuge (Beckman Coulter, Brea, CA, USA). The supernatant was recollected and filtered with a 0.22 μm syringe filter (Corning, NY, USA). The filtrated medium was later centrifuged in an Optima XPN centrifuge using a SW 32 Ti rotor (Beckman Coulter, Brea, CA, USA) at a speed of 125,000× *g* for 90 min at a temperature of 4 °C. All tubes were sealed and kept on ice. After ultracentrifugation, the supernatant was removed, and the pellet was resuspended in sterile PBS. To be sure that exosomes were correctly isolated, protein concentration was measured at 280 nm in a NanoDrop 1000 Spectrophotometer (Thermo Scientific, Waltham, MA, USA) with bovine serum albumin (BSA) as a standard in a previously prepared calibration curve. Once the presence of exosomes was verified, the samples were stored at −80 °C until use.

### 2.4. Exosome Characterization

Isolated exosomes were characterized by their size and zeta potential and the presence of the exosomal marker CD63. Average size and zeta potential characterization were performed by dynamic light scattering (DLS) with Zetasizer Nano ZSP equipment (Malvern Instruments, Worcestershire, UK). Previously isolated HepG2, HDFa and RIN-m cell-derived exosomes resuspended in PBS were used and adjusted to present a total protein concentration between 650 and 1000 µg/mL, for which they were diluted in filtered Milli-Q water. The samples were introduced in quartz cuvettes for size and zeta potential measurements. The measurement was conducted at 25 °C and at an angle of 175°. The exosomal marker CD63 was detected using the ExoELISA-Ultra CD63 Kit (System Biosciences, Palo Alto, CA) as per the manufacturer’s instructions. The CD63 standard solution provided by the kit was used to build the calibration curve.

### 2.5. Human Insulin Labeling and Detection

Human recombinant insulin was labeled with fluorescein isothiocyanate (FITC) according to the procedure proposed by Shah et al. [19]. A FITC solution in DMSO (5 mg/mL) was added dropwise with slow stirring to human insulin (15 mg/mL) in a 0.1 M bicarbonate buffer at pH 9.5. The molecular ratio of FITC to insulin selected according to the results presented in this protocol was 3:1. The reaction mixture was kept at room temperature (RT) for 150 min with continuous stirring and protected from light, then the mixture was incubated for 30 min at RT without stirring. To separate free FITC from FITC–insulin conjugates, the reaction mixture was passed through PD-10 Desalting G25 columns (GE Healthcare, Buckinghamshire, UK) and eluted using 10 mM PBS at pH 7.4. The eluent was collected in fractions of 0.5 mL and analyzed in a NanoDrop 1000 Spectrophotometer (Thermo Scientific, Waltham, MA, USA) to measure the absorbance at 280 nm and to determine in which fractions the FITC–insulin conjugates were present. Protein quantification was performed by a BCA protein assay kit and was read in a Synergy HT MultiDetection Microplate Reader (BioTek instruments Inc., Winooski, VT, USA). Once the FITC–insulin conjugates were detected, the samples were stored at −20 °C and protected from light until their use.

The fluorescence of several concentrations of FITC–insulin conjugates ws measured on a Synergy HT MultiDetection Microplate Reader (BioTek instruments Inc., Winooski, VT, USA). Human FITC–insulin standards were purchased from Sigma-Aldrich (Saint Louis, MO, USA) and used in known concentrations for quantification purposes. To establish a correlation between relative fluorescence units (RFU) and protein concentration, standard dilutions were compared with our FITC–insulin conjugate dilutions in black polystyrene 96-well microplates (Corning, NY, USA). The fluorescence assay was performed at a maximum excitation and emission of 485/528 nm.

### 2.6. FITC–Insulin Exosome Loading

To evaluate the loading properties of the three types of cell-derived exosomes with human insulin and the possible influence on their structure and physicochemical properties, the previously obtained FITC–insulin was loaded by electroporation into HepG2-, HDFa- and RIN-m-derived exosomes. For this, 400 µg/mL of the cell-derived exosomes were washed with 2 mg/mL of FITC–insulin and sterile PBS to reach a mixture final volume of 400 µL. The same concentrations were applied for HepG2, HDFa- and RIN-m-derived exosomes. The loading experiment was performed under two different loading conditions to evaluate the optimal encapsulation parameters: (a) incubation at room temperature (RT) for 1 h, (b) electroporation at 200 V and 50 µF in 0.2 cm Invitrogen electroporation cuvettes (Invitrogen, Carlsbad, CA, USA). Electroporation conditions were selected according to previous reports generated by our research group [20]. For this, sample mixtures were electroporated in a Gene Pulser Xcell Electroporation System (Bio-Rad Laboratories, Inc., Hercules, CA, USA) and subsequently incubated at 37 °C for 1 h for membrane regeneration. The loading mixtures were then transferred to an Amicon Ultra-0.5 mL 100,000 kDa device and centrifuged using an Eppendorf 5417R centrifuge at 14,000× *g* for 5 min. For a total isolation of the electroporated and loaded exosomes, the ultrafiltration procedure was repeated four times by washing the retentate with 300 µL of 10 mM filtered PBS. The remaining retentate was recovered and resuspended with 200 µL of the same PBS solution and kept for further use.

### 2.7. Evaluation of Exosome Loading Efficiency

To determine the loading efficiency of the electroporation assay, black polystyrene 96-well microplates (Corning, NY, USA) were prepared with 50 µL of electroporated exosomes and their collected supernatants, and immediately measured in a Synergy HT MultiDetection Microplate Reader (BioTek instruments Inc., Winooski, VT, USA), according to our FITC–insulin fluorescence assay parameters (excitation/emission = 485/528 nm) to evaluate the loading efficiency. FITC–insulin and non-loaded exosomes were used as negative and positive controls, respectively.

### 2.8. In Vitro Exosome Cellular Uptake and Evaluation of FITC–Insulin Cellular Internalization

HepG2, HDFa and RIN-m cells were used for in vitro uptake assays. The cells were maintained in DMEM supplemented with 10% of exosome-free FBS for at least 24 h before adding the electroporated exosomes.

Black polystyrene 96-well microplates (Corning, NY, USA) were prepared with a 50 µL suspension containing 1 ×$\;10^{4}$ cells per well and were incubated at 37 °C in a humidified 5% CO_2_ atmosphere for 24 h to allow correct attachment of the cells. After 24 h, 50 µL of exosomes was added to each well and incubated at 37 °C in a humidified atmosphere. After 6 and 24 h, the wells were washed three times with 100 µL of 10 mM PBS to remove the excess exosome in the extracellular medium. The cells were fixed with a 4% formaldehyde solution (Sigma-Aldrich, Saint Louis, MO, USA) for 20 min at room temperature and protected from light. Subsequently, the fluorescence intensity was measured in a Synergy HT MultiDetection Microplate Reader (BioTek instruments Inc., Winooski, VT, USA) according to the previous parameters used in the FITC–insulin fluorescence assays (i.e., excitation/emission = 485/528 nm). Fluorescence images of the cells were captured using a TwinCam (Cairn Research, Faversham, UK) coupled to a Nikon Eclipse Ti microscope (Nikon Instruments, Tokyo, Japan) with Nikon S Fluor 10×/0.5 and 20×/0.75 objective lenses.

### 2.9. In Vitro Evaluation of Glucose Regulation Levels of Exosome-Encapsulated Human Insulin

HepG2, HDFa and RIN-m cells were prepared in a 50 µL suspension containing 1 ×$\;10^{4}$ cells per well in black polystyrene 96-well microplates (Corning, NY, USA) and were incubated at 37 °C in a humidified 5% CO_2_ atmosphere for 24 h. The cells were then transferred to a 30 mM D-(+)-glucose enriched DMEM medium and treated with 10 µg/mL of FITC–insulin loaded exosomes. Non-treated and 10 µg/mL free FITC–insulin-treated cells were used as controls.

After 24 h, the supernatants were collected and the glucose levels were quantified using the dinitrosalicylic acid (DNS) method, which is widely used to estimate the concentration of reducing sugars in a variety of samples [21]. The method is based on the reduction of 3,5-dinitrosalicylic acid to the colored 3-amino-5-nitrosalicylate with maximum absorption at 540 nm. The optical absorbance is directly proportional to the amount of reducing sugars [22]. The DNS reagent was prepared according to the protocol described by Miller [21,23].

Glucose standards were prepared by serial dilutions of 30 mM D-glucose in ultrapure water to build the calibration curve. The reaction mixture was prepared by adding 60 µL of each sample to 120 µL of the DNS reagent. The mixture was vortexed and incubated in a controlled temperature water bath at 95 °C for 5 min. Subsequently, the mixture tubes were cooled at 4 °C for a few minutes until the samples reached room temperature. Afterwards, a 96-well polypropylene microplate (Corning, NY, USA) was prepared by adding 260 µL of ultrapure water and 36 µL of each reaction mixture. The absorbance of the samples was recorded at 540 nm against a reagent blank by a Synergy HT Multi-Detection Microplate Reader (BioTek instruments Inc., Winooski, VT, USA).

### 2.10. Data Analysis

All experiments were performed at least in triplicate; the data presented in this work correspond to the average of these replicates with the corresponding estimated error. Data analysis was performed using the statistical software Minitab 18 (Minitab, Inc., 2017, State College, PA, USA). All data were analyzed by a one-way ANOVA coupled with Fisher’s LSD test, and a *p*-value < 0.05 was established to determine significant differences.

## 3. Results and Discussion

### 3.1. Exosome Isolation and Characterization

**Figure 1:**
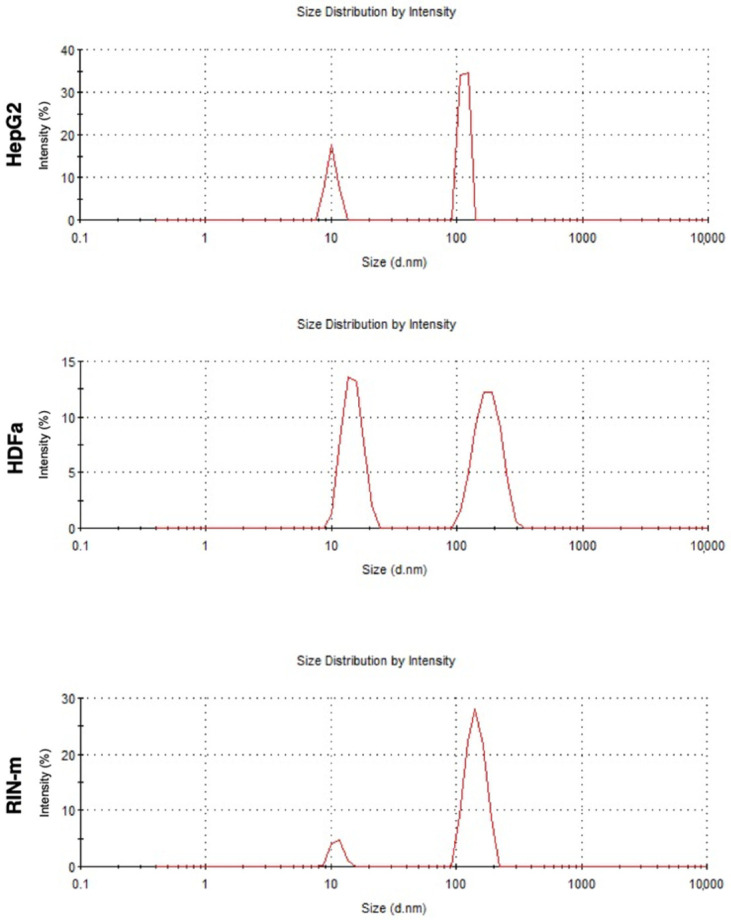
DLS size characterization for HepG2-, HDFa- and RIN-m- derived exosomes.

After isolation, exosomes obtained from HepG2, HDFa and RIN-m cells presented a narrow size distribution (Figure 1), with average sizes of 153 ± 64, 107 ± 101 and 137 ± 127 nm for the HepG2, HDFa and RIN-m cell-derived exosomes, respectively. Likewise, the measured zeta potential values were around −8.00 ± −7.18, −2.65 ± −2.07 and −4.13 ± −2.25, respectively.

The DLS and zeta potential results agree with those of previous studies performed with adherent cell-derived exosomes, showing an approximate size average of 136 ± 19 nm for human embryonic kidney cells (HEK 293T), endothelial colony-forming cells (ECFC) and human mesenchymal stem cells (MSC) [24], which are also comparable with other reported studies [25]. Furthermore, HepG2 cell-derived exosomes have been characterized in several studies, obtaining a similar average size, which has been reported to be slightly variable, depending on the cargo [26]. Regarding the zeta potential measurements, the results showed negative values between −8.00 mV and −2.65 mV, which are attributed to the negatively charged anionic phospholipid membrane [24,26]. As pancreatic beta cells, RIN-m cells present negatively charged phospholipids, namely phosphatidylserine and phosphatidylinositol, which have been reported to be in a dynamic state during insulin exocytosis, playing an important role in enhancing the fusion of lipid layers [27]. It is reported that vesicles with zeta potentials that vary between −30 mV and +30 mV tend to aggregate, although their stability varies depending on the particle type, nature, or cell culture. The stability and ability of exosomes to successfully deliver signals and compounds depends on the zeta potential, the pH and the ionic strength of the surrounding biological fluid or tissue [28]. Even though the cells used in this study were morphologically heterogenous, their exosomes showed comparable sizes and zeta potential values, with no indication of aggregation. To validate the isolation of exosomes, the presence of CD63 was corroborated in all three samples [29,30].

### 3.2. Human Insulin Labeling and Detection

**Figure 2:**
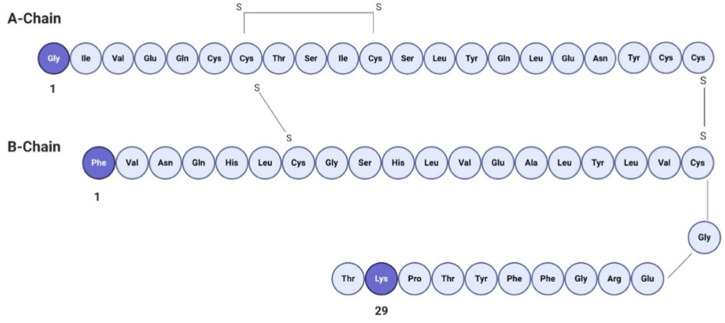
Primary structure of human insulin. Available amine sites for FITC labeling are shown in darker shades.

FITC is an amine reactive fluorophore used to fluorescently label proteins through the amine groups in the protein of interest, displaying a maximum absorption excitation at 495 nm and emission at 525 nm in the visible range of the spectrum [31]. FITC forms a covalent bond between its isothiocyanate group and the primary and secondary amine groups of biomolecules [32]. FITC (MW 389.4 Da) labeling of human insulin (MW 5807.57 Da) provides mono-, di- and tri-conjugates of insulin, since insulin presents three reactive amine sites [32]. It has been reported that conjugation at different sites affects the biological activity of insulin, as presented in Figure 2. Mono-conjugated FITC–insulin (bonded to B1 site) has the same biological activity as native insulin. However, when FITC is tagged at the A1 position, the biological activity shows a 10% decrease. Furthermore, di- and tri-conjugates show a 100% decrease in the biological activity in comparison with native insulin [33].

In this work, the presence of FITC-insulin was confirmed and characterized by a BCA protein assay kit and revealed in a Synergy HT MultiDetection Microplate Reader. A total of 26.93 mg/mL of FITC–insulin was quantified as the result of the labeling procedure, with an expected molecular ratio of 3:1 (MW = 6196.97 Da), according to the results obtained by Shah et al. [19]. It has been reported that lipophilicity and molecular weight play a crucial role in the passive diffusion and permeability of drug molecules through a variety of biological membranes [34]. The presence of FITC–insulin mono-conjugates showed enhanced permeability in comparison with tri-conjugates (MW = 6975.77 Da). As is known, FITC is much more hydrophobic than insulin [35]. Consequently, the significant lipophilicity of FITC–insulin will trigger an increase in the permeability of human insulin into exosomes. In addition, it has been reported that lipophilic compounds such as polyphenols are capable of causing interdigitation. An interdigitated structure is formed when the acyl chains of phospholipids insert into the opposite phospholipid layer, such as the exosomal membrane. This process causes the failure of the fluidity gradient of the acyl chains limiting the freedom of movement of the lipid layer, forming the interdigitated structure. In this regard, the molecular ratio of the incorporated molecule of interest is essential for the formation of this stable structure [36]. It is important to mention that to determine FITC–insulin concentrations, a calibration curve was previously prepared using dilutions of the purchased standard with a known concentration, yielding an extinction coefficient of 1248.8 with a R^2^ value of 0.9919.

### 3.3. Evaluation of FITC–Insulin Loading Efficiency in Exosomes

**Figure 3:**
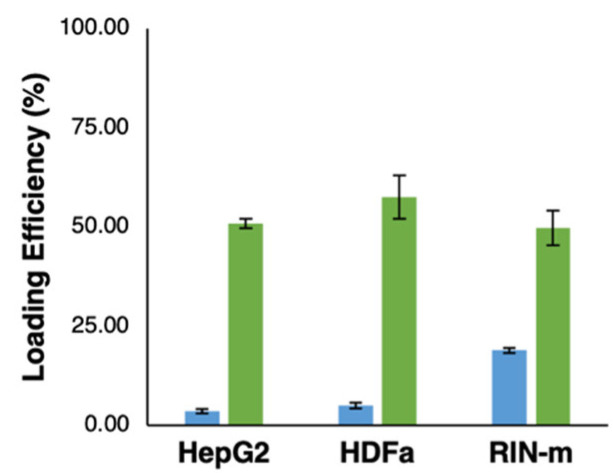
Loading efficiency of FITC–insulin (2 mg/mL) in HepG2-, HDFa- and RIN-m -derived exosomes. The graph shows the loading efficiency obtained with the incubation for 1 h at room temperature (RT) treatment (blue bars) and the electroporation at 200 V and 50 µF treatment (green bars).

The total concentration of FITC–insulin loaded in each type of cell-derived exosome was calculated using the results obtained by a fluorescence measurement, according to our FITC–insulin fluorescence assay parameters (excitation/emission = 485/528 nm), as shown in Table 1. The loading properties of HepG2-, HDFa- and RIN-m-derived exosomes were evaluated through the previously mentioned treatments. The efficiency showed significant differences (*p* < 0.05) between the loading conditions, as shown in Figure 3. The highest loading efficiency was detected with the electroporation at 200 V and 50 µF treatment, with a total concentration of exosomes of 400 µg/mL (50.75 ± 1.2%, 57.42 ± 5.47% and 49.70 ± 4.32% for HepG2, HDFa and RIN-m exosomes, respectively). Although the loading efficiencies present statistically significant differences, the efficiencies are apparently in the same range of values, a fact that may be associated with their similar particle size and zeta potential, as was previously mentioned in this work. These loading efficiencies are visibly higher than the efficiencies in the RT treatments (3.47 ± 0.56%, 4.98 ± 0.71% and 18.84 ± 0.62% for the HepG2, HDFa and RIN-m exosomes, respectively). As expected, the electroporation method presented remarkably high efficiency. As has been reported in previous studies, voltages between 140 V and 200 V show a higher encapsulation efficiency compared with lower voltages. On the other hand, variation of the capacitance (0–400 µF) does not compromise the efficiency of the electroporation procedure [37]. To exemplify this, Tian et al. [38] previously reported a similar drug loading experiment using human breast cancer-derived exosomes and doxorubicin (Dox), a well-known chemotherapeutic agent. To load the exosomes with Dox, they also tested two different methods: electroporation at 350 V and a RT co-incubation at 37 °C for 30 min without electroporation. According to their results, Dox was hardly detectable in the RT incubation sample, demonstrating the essential role of electroporation in drug loading [38]. Moreover, the electroporation loading efficiencies were higher than some already reported for the encapsulation of compounds with a lower molecular weight. In addition, Kim et al. [39] obtained loading efficiencies of 28.29% after a 1000 kV electroporation in paclitaxel encapsulation. Regarding the possible effect of the electroporation protocol on insulin’s stability, several studies have confirmed that this electrical protocol is safe and does not fragment the insulin molecule. Moreover, the same studies suggested that coupling electroporation with electroosmosis techniques has helped to achieve a greatly increased transport for insulin in in vivo models [40].

### 3.4. In Vitro Evaluation of Cellular Exosome Uptake and Cellular Incorporation of FITC–Insulin

**Figure 4:**
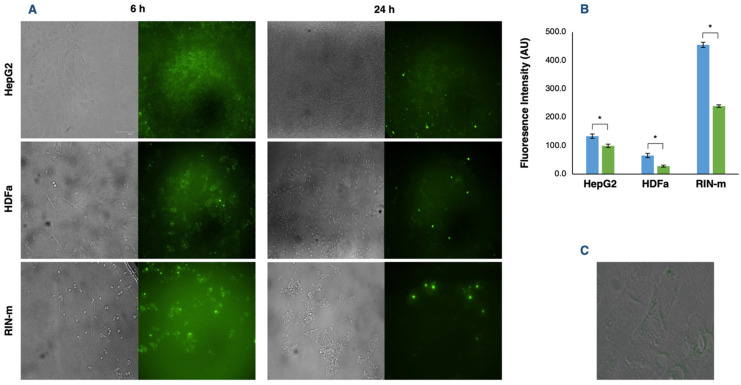
Exosome uptake by HepG2, HDFa and RIN-m cells. (**A**) Fluorescence microscopy images of HepG2, HDFa and RIN-m cells incubated with their respective exosomes loaded with FITC–insulin after 6 and 24 h. (**B**) Fluorescence intensity quantification after 6 (blue bars) and 24 h (green bars). The bars represent the mean intensity and the standard error. * Significant differences between treatments (Student’s *t*-test; *p* < 0.05). (**C**) Fluorescence microscopy image of an HDFa cell 6 h after exosome co-incubation. Loaded exosomes can be visually located inside the cytoplasm, dispersed between the cytosolic side of the plasma membrane and the perinuclear region.

Cellular uptake and, consequently, the internalization of compounds of interest is an essential point to be considered in the search for new drug delivery techniques. Therefore, the fluorescence intensity of FITC–insulin-loaded exosomes was measured to estimate their in vitro cellular uptake. The results provided by the FITC–insulin fluorescence signal and the captured images of the cells 6 and 24 h after adding the exosomes showed significant differences among the three cell types, as well as time-dependent differences in the cellular uptake of the recipient cells (*p* < 0.05) (Figure 4A). Fluorescence intensity reached the highest point 6 h after adding the exosomes in all cell types, obtaining a remarkably higher outcome in RIN-m cells (*p* < 0.05), followed by HepG2 and HDFa cells, with a visibly lower incorporation of FITC–insulin-loaded exosomes (*p* < 0.05). This elevated internalization capacity of RIN-m cells could be justified by the dual secretory exocrine and endocrine characteristics of the pancreatic beta cells [41]. Thus, HDFa cells could present the lowest uptake capacity, mainly because dermal fibroblasts are relatively ‘passive’ cells that are responsible for the synthesis and remodeling of extracellular matrix proteins [42].

After 24 h, there was an appreciable decrease in the detected fluorescence intensity and, in consequence, in the exosome uptake of all cell lines, showing significant differences (*p* < 0.05) in the exosome uptake at 6 and 24 h (Figure 4B). These results are congruent with previous time-dependent uptake assays. Specifically, it has been reported that exosomes can be detected in a small number (5%) of the cells at 5 min. At 30 min, more exosomes were internalized by the cells, accumulating in the perinuclear region [43]. Furthermore, from 30 min to 3 h, a significant number of exosomes accumulated near the nucleus, which can be visualized as bright spots (Figure 4C). Tian et al. [43] also emphasized that although exosomes are continually transported to the perinuclear regions, not all the fluorescent exosomes are accumulated in this region after 24 h, suggesting that the exosome components and cargo were probably sorted to the perinuclear region and the fluorophore, followed by a process of degradation and recycling with other membrane proteins and lipids. Moreover, Obregon et al. [44] demonstrated that dendritic-derived exosomes were mostly internalized by epithelial cells in the early hours (1–6 h) and decreased by over 24 h under similar explanations. For instance, after binding the target cells, exosomes can follow two different pathways: exosomes could remain associated with the cell surface, as has been observed in follicular dendritic cells [45], or they could be internalized by endocytosis.

Regarding these mechanisms, tetraspanins play an essential role by interacting among themselves and with other transmembrane and cytosolic proteins [46]. In several studies, it has been reported that exosomes are highly enriched in tetraspanins such as CD63, CD81, CD82 and CD37 [47]. However, the tetraspanin internalization mechanism is still being researched and discussed. According to the endocytosis mechanism, it may involve clathrin-coated vesicles, whose diameter is similar to or larger (~150 nm) than that of exosomes, because of specific receptor activation [48].

Furthermore, according to recent publications, engineered exosomes loaded by electroporation has shown to be internalized by HepG2 cells via Scavenger receptor class B type 1 (SR-B1) receptor-mediated endocytosis [26]. The SR-B1 receptor, which is highly expressed in hepatic carcinoma cells, has been associated with the mediation of phospholipid transfer between high-density lipoprotein (HDL) and the cell membrane [49]. Furthermore, Zanotti et al. [50] confirmed exosomal internalization by muscle-derived fibroblasts and suggested that cellular uptake of the fibroblast-derived exosomes may take place via phagocytosis or micropinocytosis rather than plasma membrane fusion, as an energy-dependent process confirmed by a succinate dehydrogenase (MTT) activity assay.

### 3.5. In Vitro Evaluation Glucose Regulation Levels of Exosome-Encapsulated Human Insulin

**Figure 5:**
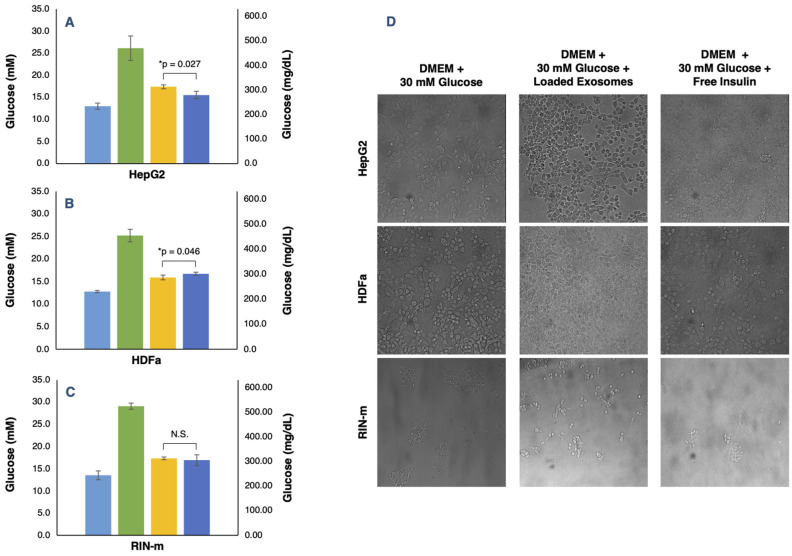
In vitro determination of glucose concentrations in HepG2, HDFa and RIN-m cell lines treated with DMEM + 10% FBS (light blue bars), DMEM + 30 mM glucose (green bars), DMEM + 30 mM glucose + insulin-loaded exosomes (yellow bars) and DMEM + 30 mM glucose + free insulin (dark blue bars). (**A**) HepG2-derived exosomes and free insulin treatments were significantly different (*p* < 0.05), showing lower glucose levels in the DMEM + 30 mM glucose + free insulin treatment. (**B**) HDFa-derived exosomes and free insulin treatments presented significant differences (*p* < 0.05), showing lower glucose levels in the DMEM + 30 mM glucose + insulin-loaded exosome treatment. (**C**) Loaded RIN-m derived exosomes and free insulin treatments did not present significant differences (*p* < 0.05), showing equal glucose concentration levels. (**D**) Morphological and cell population variation for the DMEM + 30 mM glucose, DMEM + 30 mM glucose + insulin-loaded exosomes and DMEM + 30 mM glucose + free insulin treatments in the HepG2, HDFa and RIN-m cell lines. Brightfield images were captured 24 h after starting each treatment. The error bars in the graphs represent the mean concentration plus the standard error. * Significant differences between treatments (Student’s *t*-test; *p* < 0.05); N.S. represents non-significant differences.

In vitro evaluation of the glucose regulation levels of the FITC–insulin-loaded exosomes and free FITC–insulin at the same concentration was performed. The results showed that HepG2-derived exosomes had an appreciable effect on glucose level regulation by decreasing the glucose concentration in a hyperglycemic medium. Particularly, HepG2 cells treated with free insulin presented lower glucose levels; in consequence, the free insulin treatment was more effective than the exosome-mediated insulin delivery, showing significant differences (*p* < 0.05), as shown in Figure 5A. Additionally, morphological and cell population changes were observed as result of the different treatments. As shown in Figure 5D, we can appreciate the cytoplasmic granules in HepG2 cells exposed to a hyperglycemic medium, a fact that indicates cellular stress and toxicity [51]. Moreover, in HepG2 cell cultures, we noted signs of cytoplasmic stress granules but only a slightly visible incidence in the cells treated with the DMEM + 30 mM glucose + free insulin. Thus, HepG2 cells, as a liver cancer cell line, are natural insulin target cells because of their essential role in the glucose transport across the plasma membrane associated with the abundant membrane expression of the GLUT2 glucose transporter [52]. Several studies have suggested that the exposure of HepG2 cells to elevated glucose levels leads to an increase in tyrosine phosphorylation of the insulin receptor (IR) [53]. According to the obtained results, free insulin administration ameliorates hyperglycemia, probably by stimulating IR and, consequently, promoting cellular glucose metabolism. Therefore, in this case, insulin-loaded exosomes did not enhance the rapid decrease in glucose levels by adding free insulin only. However, there was a significant effect of the insulin-loaded exosomes, showing that it could be a promising delivery vehicle that must be optimized.

For their part, HDFa-derived exosomes produced a visible effect in the regulation of the levels of glucose by decreasing the glucose concentration in a hyperglycemic medium. Unlike the HepG2 cells, HDFa cells treated with insulin-loaded exosomes were shown to reduce glucose levels considerably more than the free insulin treatment, also presenting statistically significant differences (*p* < 0.05) (Figure 5B). As shown in Figure 5D, cytoplasmic granules in the HDFa cell culture exposed to a hyperglycemic medium could be noted but, on the contrary, the signs of cellular stress were less visible in the cells treated with the DMEM + 30 mM glucose + loaded exosome medium. In this case, it is well known that skin fibroblasts contain both insulin and insulin growth factor I (IGF-I) receptors, which are probably hybrid receptors [54]. Although skin fibroblasts are not classic target tissues, some abnormalities in insulin binding and receptor autophosphorylation found in fibroblasts have been associated with mutations in the insulin receptor gene [55]. In this work, we have demonstrated that exosome-mediated delivery of insulin could become an effective tool in comparison with the administration of free insulin, but the precise point in the glucose metabolism and transport of HDFa cells that enhances the insulin delivery and, consequently, effectively decreases hyperglycemia are still unclear.

Finally, the RIN-m-derived exosomes also had an appreciable effect on the regulation of the levels of glucose by decreasing the glucose concentration in a hyperglycemic medium. Unlike the other cell lines, RIN-m cells treated with insulin-loaded exosomes and with free insulin had the same effect on the glucose levels of the extracellular medium. Both treatments did not present significant differences (*p* < 0.05), as shown in Figure 5C. In addition, in Figure 5D, cytoplasmic stress granules were not noted in RIN-m cells in the high-glucose medium, probably because the short-term high glucose culture potentiates and improves pancreatic beta function and proliferation [56]. Furthermore, there were no appreciable signs of cellular toxicity in the RIN-m cell cultures, although a decrease in cell density in the DMEM + 30 mM glucose + free insulin treatment was observed. This effect can be attributed to the fact that RIN-m cells, as natural producers of insulin, could suffer saturation and their insulin secretory activity can be compromised if there is an excess of insulin in the extracellular medium. Therefore, insulin-loaded exosomes and free insulin produced the same effect in the RIN-m cell culture.

Nonetheless, other exosome properties must be taken into consideration. For instance, the effect of injectable human insulin generally starts 45 min after subcutaneous administration, and the duration of its effect can be expected to be between 6 and 8 h until enzymatic degradation [57]. For their part, exosomes have been considered, in a variety of studies, to be long-lasting nanocarriers whose beneficial effects have been observed weeks after their addition to the target tissue. Specifically, Aquil et al. [58] demonstrated that exosomes loaded with curcumin formulations presented an antitumoral activity at least for 7 weeks after the addition of exosomes, and also reported that loaded exosomes were stable and had an unaltered drug load at −80 °C for up to 6 months of storage. In consequence, a bioavailability and stability study for prolongated time periods could provide a better insight into the potential exosomes that could be as valuable tools for insulin delivery in pancreatic beta cells.

## 4. Conclusions

This work was based on evidence that supported exosomes as highly efficient vehicles for the delivery of therapeutic biomolecules. Therapeutic compounds such as miRNAs, chemotherapeutical molecules or natural extracts have been successfully delivered to target tissues using these naturally occurring EVs. In this work, we demonstrated that exosomes may also represent a valuable delivery system for biologically active peptides such as insulin. Here, cell-derived exosomes were isolated and loaded with human insulin to be internalized by the recipient cells and to induce a biological effect in glucose transport and metabolism. Human insulin was encapsulated into the exosomes by electroporation, with a high efficiency compared with other reported insulin encapsulation methods and nanocarriers. Considering that insulin is a peptide hormone with a relatively high molecular weight, the fact that it can be encapsulated while conserving its unaltered biological function indicates a promising result. Insulin-loaded exosomes were internalized by their respectively donor cells and were able to promote and enhance the transport and metabolism of glucose in hyperglycemic environments. This evidence suggests a potential novel tool for a long-lasting, stable and non-cytotoxic insulin delivery technology. The World Health Organization estimates that the 8.5% of the global population was diagnosed with diabetes in 2014, and 2.2 million deaths were directly related to high glucose levels in 2016. These data reflect the necessity of continuing to search for and find novel therapies and treatments for Type 1 and 2 diabetes. Novel cell-specific techniques for exosome delivery may not only represent a drug delivery tool but could also present a means of targeting biomarkers for preventive medicine and for the control of disease progression. However, current exosome isolation and purification protocols are still a time-consuming process with relatively low outcomes. Therefore, new scalable, effective and economically sustainable processes are required for large-scale exosome isolation and engineering. Nevertheless, nanotechnology is rapidly developing and, in the near future, will surely provide a solution to solve some of the existing challenges in exosome engineering and could lead the way to new personalized therapies.

## Figures and Tables

**Table 1:** FITC–insulin concentration in loaded HepG2-, HDFa- and RIN-m- derived exosomes in the room temperature (RT) and electroporation at 200 V and 50 µF treatments. After both loading procedures, the samples were incubated at 37 °C for 1 h to allow membrane regeneration before analysis.

Cell-Derived Exosomes	Room Temperature (mg/mL)	Electroporation (mg/mL)
HepG2	0.07 ± 0.08	1.04 ± 0.99
HDFa	0.09 ± 0.05	1.10 ± 1.19
RIN-m	0.38 ± 0.12	0.91 ± 1.09

## Data Availability

Data is contained within the article.
